# Energy Harvesting over Rician Fading Channel: A Performance Analysis for Half-Duplex Bidirectional Sensor Networks under Hardware Impairments

**DOI:** 10.3390/s18061781

**Published:** 2018-06-01

**Authors:** Tan N. Nguyen, Tran Hoang Quang Minh, Phuong T. Tran, Miroslav Vozňák

**Affiliations:** 1 Wireless Communications Research Group, Faculty of Electrical and Electronics Engineering, Ton Duc Thang University, Ho Chi Minh City, Vietnam; nguyennhattan@tdt.edu.vn; 2 Faculty of Electrical Engineering and Computer Science, VŠB-Technical University of Ostrava, 17. Listopadu 15/2172, 708 00 Ostrava, Czech Republic; miroslav.voznak@vsb.cz; 3 Optoelectronics Research Group, Faculty of Electrical and Electronics Engineering, Ton Duc Thang University, Ho Chi Minh City, Vietnam; tranhoangquangminh@tdt.edu.vn

**Keywords:** half-duplex, relay networks, wireless energy harvesting, time-switching, Rician fading channel, hardware impairment

## Abstract

In this paper, a rigorous analysis of the performance of time-switching energy harvesting strategy that is applied for a half-duplex bidirectional wireless sensor network with intermediate relay over a Rician fading channel is presented to provide the exact-form expressions of the outage probability, achievable throughput and the symbol-error-rate (SER) of the system under the hardware impairment condition. Using the proposed probabilistic models for wireless channels between mobile nodes as well as for the hardware noises, we derive the outage probability of the system, and then the throughput and SER can be obtained as a result. Both exact analysis and asymptotic analysis at high signal-power-to-noise-ratio regime are provided. Monte Carlo simulation is also conducted to verify the analysis. This work confirms the effectiveness of energy harvesting applied in wireless sensor networks over a Rician fading channel, and can provide an insightful understanding about the effect of various parameters on the system performance.

## 1. Introduction

Wireless sensor networks (WSNs) are subject to the constraint of energy storage at each mobile node [1,2]. Saving on energy consumption or extending the battery life for sensor nodes has become an important research issue in wireless sensor networks. Recently, energy harvesting (EH) has attracted enormous attention from researchers as a promising cost-effective technique to maximize energy efficiency of a wireless network [3,4,5], especially in wireless sensor networks [6,7,8,9]. Various energy harvesting sources have been studied, such as natural sources (solar [10], wind [11], thermal [12], etc.), strongly coupled magnetic resonances [13], etc. Among them, radio frequency (RF) energy from the ambient transmitters is the most popular source for energy harvesting since it can be received more effectively from RF signals [4]. Because RF signals can convey both energy and information simultaneously, an RF-based energy harvesting technique, called simultaneous wireless information and power transfer (SWIPT), is becoming a more and more promising research topic for WSNs [14,15]. The idea of SWIPT was first introduced in 2008 in the seminal paper of Varshney [16], in which authors proposed an ideal receiver design that is able to simultaneously observe the information and extract power from the same received signal. Zhou et al. [17] connected Varshney’s idea to practice by proposing two realizable receiver architectures design: time switching (TS) and power splitting (PS). The performance analysis of these two EH protocols was conducted rigorously by Nasir et al. [18]. The authors derived analytical expressions for the outage probability and the ergodic capacity of one-way amplify-and-forward (AF) relay networks over a Rayleigh fading channel.

The applications of SWIPT to wireless sensor networks have been investigated in recent works. In [7], the authors proposed a distributed iteration algorithm to solve an energy-efficient cooperative transmission problem for SWIPT in clustered WSNs. In [9], the authors employed wireless energy harvesting techniques and realistic energy converters in dense and often randomly deployed WSNs, and quantified the potential energy gains that can be achieved in the network. Peng et al. [19] considered a wireless energy harvesting two-way relay network (TWRN) using power splitting protocol, where the effects of practical hardware impairments were taken into consideration. Mouapi and Hakem [20] proposed a new approach to defining the specifications of a stand-alone wireless node based on an RF EH system and implemented a hardware circuit to illustrate their energy optimization method. The performance of wireless powered sensor networks for Internet of Things (IoT) application was studied in [4], where the authors proposed the optimal power allocation to maximize the system throughput and also derived the closed-form of that solution. However, in those papers, the channel gains are assumed either to be constant or to be a Rayleigh distributed random variable.

Wireless communication systems in the real radio environments are not only affected by the short-term fading (multipath), but are also subject to the shadowing effects. The vital issue in studying the performance of energy-harvesting-based wireless networks in these conditions is the outage probability analysis. An important statistical characteristic that can describe the behavior of the wireless channels is the probability density function (PDF) of the signal-to-noise-ratio (SNR) at the receiver output. To derive this PDF for different radio propagation environments is sometimes a difficult mathematical task, especially for complicated channel models such as Nakagami-m or Rician channels. In fact, not only the papers we mentioned about SWIPT for WSN, but most of the other results on outage performance up to now [21,22] also focus on Rayleigh fading channels, where we can exploit the fact that the square of channel gain magnitude is exponentially distributed.

As a general comment, it is noted that very few publications about energy harvesting for Rician fading channel exist in the open technical literature. In fact, together with Rayleigh channel, Rician channel should be also considered as one of the small-scale fading models for WSNs, especially for the cases of relatively short range power transfer distance and with existence of a strong line-of-sight (LOS) path [23]. Recently, Zhao et al. [24] have derived the capacity expressions for wireless powered communication systems over a Rician fading channel. However, there is no relay in this study. The source directly harvests the energy from the power beacon. In [25], the authors provided the throughput analysis of relay networks with two energy harvesting protocols (continuous and discrete) over a Rician fading channel, but were only limited in the case of perfect hardware. Furthermore, the paper only provided the integral form of the throughput for the continuous case, so it is not a computationally friendly result. Mishra et al. [26] did provide impressive results on joint optimization of power allocation, power splitting for EH, and relay placement for SWIPT over a Rician channel. Nevertheless, they only use an approximation expression of outage probability to formulate the optimization problem, and did not consider the hardware impairment at the nodes. In practice, the transceiver hardware is imperfect due to phase noise, I/Q (In-phase/Quadrature) imbalance and amplifier nonlinearities [27]. The modeling of hardware impairment in system performance analysis has been presented in many works, for instance, in [28], where the authors analyzed the performance of dual-hop relaying systems in hardware impairment condition, in terms of the capacity, throughput and symbol error rate (SER). We also proposed and evaluated an energy harvesting-based spectrum access model in cognitive radio network with hardware impairment [29]. Again, these works about hardware impairment considered the Rayleigh channels only.

In our current work, we tackle the problem of investigating the performance analysis of SWIPT for WSN over a Rician fading channel, which takes into account the hardware impairment at source and relay nodes. Specifically, we consider an AF two-way half-duplex energy harvesting relay network model suffering from hardware impairments at all nodes over the Rician fading channels. The exact analytical expressions of the achievable throughput, outage probability, and the exact-form expression for the PDF of SNR at each destination node of a half-duplex AF bidirectional wireless sensor networks over a Rician fading channel are derived rigorously. The main contributions of this paper can be described in more details as follows:

- The exact form expression of outage probability and achievable throughput at each destination node with imperfect hardware and in Rician fading environment are derived mathematically.
- We derive the exact-form cumulative distribution function (CDF) of the SNR at each destination node, and use this result to derive the integral exact-form of the SER at destination nodes.
- We also conduct the asymptotic analysis and provide the approximation of all performance factors mentioned above at high $P/N_{0}$ regime.
- The analytical results are all confirmed by Monte Carlo simulations. Using the simulation results, the effect of various system parameters on the system performance is carefully studied.

The rest of this paper is organized as follows. Section 2 describes the system model and the EH protocol that is used in this paper. Section 3 provides the detailed performance analysis of the system, including exact analysis and asymptotic analysis. The numerical results to validate the analysis are presented in Section 4. Finally, conclusions are drawn in Section 5.

## 2. System Model

### 2.1. System Model Description

**Figure 1:**
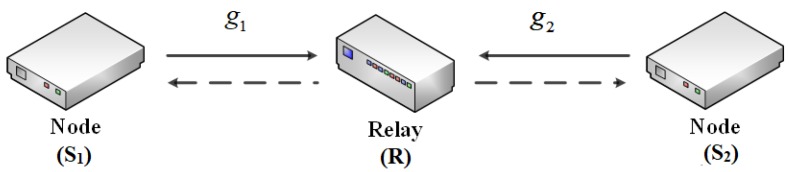
System model.

We consider a bidirectional network with two sources and one relay as illustrated in Figure 1. The transmission model follows the principles of analog network coding [30,31]. This concept is the extension of linear network coding to multihop wireless networks. Traditionally, two sources transmit their data to the relay node in two different time slots to avoid interference. However, with analog coding, the data can be transmitted simultaneously to the relay. The relay node forwards this noisy sum of two signals during the next time slot. Because each node already knows one of the signals by virtue of having forwarded it earlier, it can cancel its own part in the received signals and decode the message sent by the other source.

In our model, every terminal has only one antenna and operates in a half-duplex mode. Let $S_{1}$ and $S_{2}$ denote the first node and the second node that are going to exchange their messages, respectively, and *R* denote the relay node. We denote the channel gain between node $S_{i}$ and the relay *R* as $g_{i}$, for i=1,2. Here, both channels are assumed to be Rician fading channels and be reciprocal. In this research, we take into account the hardware impairment at both source nodes $S_{1}$, $S_{2}$, and relay node *R*. Regarding the communication between two nodes, we assume that the direct link between two nodes is very weak, and the communication relies solely with the help of relay. However, the relay has only the energy to serve their own purpose, so it needs to harvest the energy from the two nodes of interest before forwarding the information messages. As in many previous publications on two-way half-duplex channels, we assume that the two sources as well as the relay know the channel gains.

### 2.2. Energy Harvesting and Information Transfer Protocols

**Figure 2:**
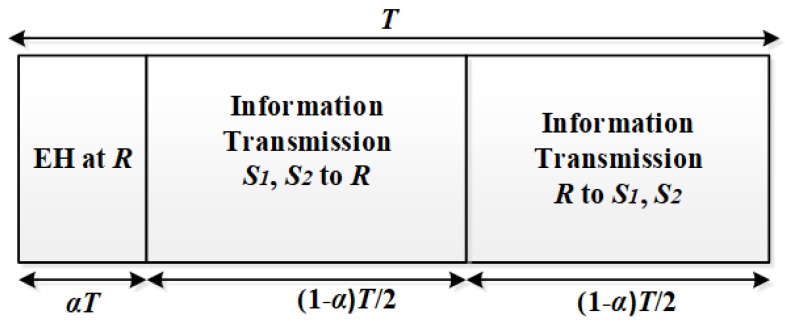
The energy harvesting and information processing in the system model.

The energy harvesting protocol for this system is illustrated in Figure 2. In this protocol, the transmission is divided into blocks of length *T*. Each transmission block consists of three time slots. In the first time slot, which has the duration of αT, the relay harvests energy from the nodes $S_{1}$ and $S_{2}$. The two remaining time slots are used for information transferring. First, $S_{1}$ and $S_{2}$ simultaneously transmit to *R* at the second time slot with the transmitted power P. Then, at the third time slot, *R* amplifies the signal that it received during the second time slot and broadcasts it to $S_{1}$ and $S_{2}$. Both the second and third time slots have the same length of (1−α)T/2.

Let *P* denote the average power transmitted by each node $S_{1}$ and $S_{2}$ during the first time slot. After the first time slot, the amount of the harvested energy at node *R* can be computed as

(1) $$E_{r}=\left(P{\left|g_{1}\right|}^{2}+P{\left|g_{2}\right|}^{2}\right)\eta \alpha T,$$

where η is the energy harvesting efficiency.

Let $s_{1}$ and $s_{2}$ denote the information-bearing symbols transmitted from $S_{1}$ and $S_{2}$, respectively. Again, assume that both $s_{1}$ and $s_{2}$ have the same average power *P*, i.e. $E\left[{\left|s_{i}\right|}^{2}\right]=P$. Thus, the received signal at *R* at the second time slot can be written as [28]

(2) $$y_{r}=\sum_{i=1}^{2}g_{i}\left(s_{i}+\eta_{i}\right)+n_{r},i=1,2,$$

where:

- $\eta_{i}$ denotes the hardware distortion noise at $S_{i}$ with zero mean and variance $\kappa^{2}P$. Here, κ is sufficient to characterize the aggregate level of impairments of the channel [1].
- $n_{r}$ is the additive white Gaussian noise (AWGN) at *R* with zero mean and variance $\sigma_{r}^{2}$.

At the second time slot, *R* amplifies the signal $y_{r}$ with an amplifying coefficient β and then retransmits it to $S_{1}$ and $S_{2}$. The received signal $y_{i}$ at node $S_{i}$ at the third time slot is

(3) $$y_{i}=g_{i}\left(\beta y_{r}+\eta_{r}\right)+n_{i},i=1,2,$$

where $\eta_{r}$ denotes the distortion noise with zero mean and variance $\kappa_{r}^{2}P_{r}$, $n_{i}$ is the additive white Gaussian noise (AWGN) at $S_{i}$ with zero mean and variance $\sigma_{i}^{2}$, for i=1,2.

According to the law of energy conservation, the total energy that node *R* uses to transmit the amplified signal to $S_{1}$ and $S_{2}$ must be equal to the energy that *R* received from $S_{1}$ and $S_{2}$ at the first time slot, $E_{r}$. Now, because the transmission duration of the relay is $\frac{(1-\alpha )T}{2}$, the average power of the signal transmitted by *R* at the third time slot can be computed as

(4) $$P_{r}=\frac{E_{r}}{(1-\alpha )T/2}=\psi \left[P{\left|g_{1}\right|}^{2}+P{\left|g_{2}\right|}^{2}\right],$$

where $\psi =\frac{2\eta \alpha }{1-\alpha }$.

In order to ensure that the transmission power at *R* is $P_{r}$, the amplifying coefficient β can be chosen as

(5) $$\beta =\frac{r}{y_{r}}=\sqrt{\frac{P_{r}}{X_{1}P+X_{1}P\kappa^{2}+X_{2}P+X_{2}P\kappa^{2}+\sigma_{r}^{2}}},$$

where $X_{i}={\left|g_{i}\right|}^{2}$ denotes the square of channel gain amplitude between $S_{i}$ and *R*.

Note that the relay node *R* does not need to estimate the hardware noise parameters as well as the individual channel gains $g_{i}$ because the denominator of Equation (5) is the received symbol power during the 2nd time slot. Now, because of the similarity between the roles of $S_{1}$ and $S_{2}$, we can focus only on node $S_{1}$. In fact, the received signal $y_{1}$ can be rewritten as

(6) $$y_{1}=\beta {\left|g_{1}\right|}^{2}s_{1}+\beta {\left|g_{1}\right|}^{2}\eta_{1}+\beta \left|g_{1}\right|\left|g_{2}\right|s_{2}+\beta \left|g_{1}\right|\left|g_{2}\right|\eta_{2}+\beta \left|g_{1}\right|n_{r}+\left|g_{1}\right|\eta_{r}+n_{1}.$$

This signal contains both messages $s_{1}$ and $s_{2}$, while only $s_{2}$ is the desired signal at $s_{1}$. Since node $S_{1}$ perfectly knows its own transmitted symbol $s_{1}$, it can eliminate the corresponding the self-interference term $\beta {\left|g_{1}\right|}^{2}s_{1}$ from $y_{1}$.

From this fact, Equation (6) can be rewritten as

(7) $$y_{1}=\underset{signal}{\underset{︸}{\beta \left|g_{1}\right|\left|g_{2}\right|s_{2}}}+\underset{noise}{\underset{︸}{\beta {\left|g_{1}\right|}^{2}\eta_{1}+\beta \left|g_{1}\right|\left|g_{2}\right|\eta_{2}+\beta \left|g_{1}\right|n_{r}+\left|g_{1}\right|\eta_{r}+n_{1}}}.$$

Therefore, the end-to-end signal-to-noise-ratio at $S_{1}$ for detection of the symbol $s_{1}$ is given by

(8) $$\gamma_{1}=\frac{E\left\{{\left|signal\right|}^{2}\right\}}{E\left\{{\left|noise\right|}^{2}\right\}}=\frac{\beta^{2}{\left|g_{1}\right|}^{2}{\left|g_{2}\right|}^{2}P}{\beta^{2}{\left|g_{1}\right|}^{4}P\kappa^{2}+\beta^{2}{\left|g_{1}\right|}^{2}{\left|g_{2}\right|}^{2}P\kappa^{2}+\beta^{2}{\left|g_{1}\right|}^{2}\sigma_{r}^{2}+{\left|g_{1}\right|}^{2}P_{r}\kappa_{r}^{2}+\sigma_{1}^{2}}.$$

After doing some algebra and using the fact that $\frac{\sigma_{1}^{2}\sigma_{r}^{2}}{P_{r}}\approx 0$, combining with Equation (6), we obtain:

(9) $$\gamma_{1}=\frac{X_{1}X_{2}}{X_{1}X_{2}a+X_{1}^{2}a+X_{1}b+c},$$

where $a=\kappa^{2}+\kappa_{r}^{2}\left(1+\kappa^{2}\right),b=\frac{\left(1+\kappa_{r}^{2}\right)}{P/\sigma_{r}^{2}},c=\frac{\left(1+\kappa^{2}\right)}{\psi P/\sigma_{1}^{2}}$ (assume that $\sigma_{1}^{2}=\sigma_{2}^{2}=\sigma_{r}^{2}=N_{0}$).

## 3. System Performance

### 3.1. Outage Probability

Since the random variable (RV) $X_{i}$ is Rician distributed for i=1,2, the probability density function (PDF) of $X_{i}$ can be found as

(10) $$f_{X_{i}}\left(x\right)=\frac{\left(K+1\right)e^{-K}}{\lambda_{i}}e^{-\frac{(K+1)x}{\lambda_{i}}}I_{0}\left(2\sqrt{\frac{K(K+1)x}{\lambda_{i}}}\right),$$

where $\lambda_{i}$ is the mean value of the RV $X_{i},i=1,2$, *K* is the Rician K-factor defined as the ratio of the power of the line-of-sight (LOS) component to the scattered components and $I_{0}\left(\cdot \right)$ is the zero-th order modified Bessel function of the first kind.

Using the equality $I_{0}\left(x\right)=\sum_{l=0}^{\infty }\frac{x^{2l}}{2^{2l}{\left(l!\right)}^{2}}$ [32], Equation (10) can be rewritten as

(11) $$f_{X_{i}}\left(x\right)=\zeta_{i}e^{-K}\sum_{l=0}^{\infty }\frac{{\left(\zeta_{i}K\right)}^{l}}{{\left(l!\right)}^{2}}x^{l}e^{-\zeta_{i}x},$$

where $\zeta_{i}=\frac{K+1}{\lambda_{i}}$.

The cumulative density function (CDF) of the RV $X_{i}$ (i=1,2) can be derived as in [33]:

(12) $$F_{X_{i}}\left(x\right)=\int_{0}^{x}f_{X_{i}}\left(t\right)dt=1-e^{-K}\sum_{l=0}^{\infty }\sum_{m=0}^{l}\frac{K^{l}\zeta_{i}^{m}}{l!m!}x^{m}e^{-\zeta_{i}x}.$$

The outage probability of this system is defined as the probability that the end-to-end SNR falls below a desired threshold—let us say, $\gamma_{th}$. In this case, it can be derived as

(13) $$P_{out\_1}=F_{\gamma_{1}}\left(\gamma_{th}\right)=\mathrm{Pr}\left(\gamma_{1}<\gamma_{th}\right)=\mathrm{Pr}\left(\frac{X_{1}X_{2}}{X_{1}X_{2}a+X_{1}^{2}a+X_{1}b+c}<\gamma_{th}\right),$$

where $\gamma_{th}=2^{2R}-1$, *R* is the source transmission rate:

(14) $$\begin{array}{c} \mathrm{Pr}\left(\frac{X_{1}X_{2}}{X_{1}X_{2}a+X_{1}^{2}a+X_{1}b+c}<\gamma_{th}\right)=\mathrm{Pr}\left\{X_{2}\left(X_{1}-\gamma_{th}X_{1}a\right)<\gamma_{th}X_{1}^{2}a+\gamma_{th}X_{1}b+\gamma_{th}c\right\},\\ =\left\{\begin{array}{c} \mathrm{Pr}\left\{X_{2}<\frac{\gamma_{th}X_{1}^{2}a+\gamma_{th}X_{1}b+\gamma_{th}c}{X_{1}-\gamma_{th}X_{1}a}\right\},\;\mathrm{if}\;1-\gamma_{th}a\geq 0,\\ 1,\;\;\;\;\;\;\;\;\;\;\;\;\;\mathrm{if}\;1-\gamma_{th}a<0. \end{array}\right. \end{array}$$

In this subsection, we assume that $1-\gamma_{th}a$ is positive, which is reasonable in practice. From Equation (14), we can derive the exact-form expression for the outage probability of the system. This is stated by the following theorem.

**Theorem** **1** (Outage probability—Exact form)**.** *The exact outage probability for node $S_{1}$ of the proposed half-duplex bidirectional wireless sensor network using time-switching energy harvesting strategy over a Rician fading channel can be expressed as*

(15) $$\begin{array}{c} P_{out\_1}=1-2\zeta_{1}e^{-2K}e^{-b_{1}\zeta_{2}{\tilde{\gamma }}_{th}}\sum_{l=0}^{\infty }\sum_{k=0}^{\infty }\sum_{m=0}^{l}\sum_{n=0}^{m}\sum_{p=0}^{n}\frac{K^{l+k}\zeta_{1}^{k}{\left(\zeta_{2}{\tilde{\gamma }}_{th}\right)}^{\frac{3m-2n+p+k+1}{2}}a^{m-n}b^{p}c^{\frac{m-p+k+1}{2}}}{l!p!\left(n-p\right)!\left(m-n\right)!{\left(k!\right)}^{2}{\left(\zeta_{1}+a\zeta_{2}{\tilde{\gamma }}_{th}\right)}^{\frac{m-2n+p+k+1}{2}}}\\ \times K_{m-2n+p+k+1}\left(2\sqrt{\left(\zeta_{1}+a\zeta_{2}{\tilde{\gamma }}_{th}\right)\zeta_{2}{\tilde{\gamma }}_{th}c}\right), \end{array}$$

*where $a=\kappa^{2}+\kappa_{r}^{2}\left(1+\kappa^{2}\right),b=\frac{\left(1+\kappa_{r}^{2}\right)}{P/\sigma_{r}^{2}},c=\frac{\left(1+\kappa^{2}\right)}{\psi P/\sigma_{i}^{2}},\zeta_{i}=\frac{K+1}{\lambda_{i}}$, and ${\tilde{\gamma }}_{th}=\frac{\gamma_{th}}{1-a\gamma_{th}}$.*

**Proof** **of** **Theorem** **1.** See Appendix A. ☐

**Remark** **1.** *The outage probability at node $S_{2}$ can also be obtained by exchanging the roles of $\zeta_{1}$ and $\zeta_{2}$ in Equation* (15)*.*

### 3.2. Achievable Throughput

With the outage probability obtained from Theorem 1, the achievable throughput of the considered system can be given by

(16) $$\tau_{i}=\left(1-P_{out\_i}\right).\frac{R}{2}.\left(1-\alpha \right),i\in \left\{1,2\right\}.$$

### 3.3. SER Analysis

In this section, we derive the formula for symbol error rate (SER) at the destination. In fact, the theoretical SER at the receiving node can be computed in terms of the end-to-end SNR at that node [34]. By taking into account the randomness of the SNR in our model, the SER at node $S_{i}$ can be derived by taking the expectation with respect to the random variable SNR:

(17) $${SER}_{i}=E\left[\omega Q\left(\sqrt{2\theta \gamma_{i}}\right)\right],i\in 1,2,$$

where $Q\left(t\right)=\frac{1}{\sqrt{2\pi }}\int_{t}^{\infty }e^{-x^{2}/2}dx$ is the Gaussian Q-function; ω and θ are constants that are specific for each modulation type—in particular, ω=1,θ=1 for Binary Phase Shift keying (BPSK) and ω=1,θ=2 for Quadrature Phase Shift Keying (QPSK). Hence, before obtaining the SER formula, the CDF of $\gamma_{i}$ is needed. Let $F_{\gamma_{i}}\left(x\right)$ be the CDF of $\gamma_{i}$. By exchanging the order of integration and changing variable, we come up with the following formula:

(18) $${SER}_{i}=\frac{\omega \sqrt{\theta }}{2\sqrt{\pi }}\int_{0}^{\infty }\frac{e^{-\theta x}}{\sqrt{x}}F_{\gamma_{i}}\left(x\right)dx.$$

Now, we can claim the following theorem on SER.

**Theorem** **2** (SER—Exact form)**.** *The exact symbol error rate for node $S_{1}$ of the proposed half-duplex bidirectional wireless sensor network using time-switching energy harvesting strategy over a Rician fading channel can be expressed as:*

(19) $$\begin{array}{c} SER_{1}=\frac{\omega }{2}-\zeta_{1}e^{-2K}\omega \sqrt{\theta }\sum_{l=0}^{\infty }\sum_{k=0}^{\infty }\sum_{m=0}^{l}\sum_{n=0}^{m}\sum_{p=0}^{n}\frac{K^{l+k}\zeta_{1}^{k}a^{m-n}b^{p}c^{\frac{m-p+k+1}{2}}}{l!p!\left(n-p\right)!\left(m-n\right)!{\left(k!\right)}^{2}}\times \\ \times \int_{0}^{\infty }\frac{e^{-\theta x-b\zeta_{2}{\tilde{\gamma }}_{th}\left(x\right)}}{\sqrt{\pi x}}K_{m-2n+p+k+1}\left(2\sqrt{\left[\zeta_{1}+a\zeta_{2}{\tilde{\gamma }}_{th}\left(x\right)\right]\zeta_{2}{\tilde{\gamma }}_{th}\left(x\right)c}\right)\frac{{\left[\zeta_{2}{\tilde{\gamma }}_{th}\left(x\right)\right]}^{\frac{3m-2n+p+k+1}{2}}}{{\left[\zeta_{1}+a\zeta_{2}{\tilde{\gamma }}_{th}\left(x\right)\right]}^{\frac{m-2n+p+k+1}{2}}}dx, \end{array}$$

*where ${\tilde{\gamma }}_{th}\left(x\right)=\frac{x}{1-ax}$.*

**Proof** **of** **Theorem** **2.** See Appendix B. ☐

### 3.4. Asymptotic Analysis

The asymptotic analysis is significant to provide further insights into the impact of hardware impairments on the network performance. It can also be used to verify the correctness of the exact analysis. In this section, the asymptotic outage probability and SER at the high SNR regime are going to be derived rigorously.

#### 3.4.1. Outage Probability

As the $P/N_{0}$ approaches infinity, it is obvious to see that the SNR in Equation (9) is asymptotically equal to

(20) $$\gamma_{1}^{\infty }=\frac{X_{2}}{aX_{2}+aX_{1}}=\frac{X_{2}}{a(X_{1}+X_{2})},$$

and, similarly,

(21) $$\gamma_{2}^{\infty }=\frac{X_{1}}{a(X_{1}+X_{2})},$$

where $a=\kappa^{2}+\kappa_{r}^{2}\left(1+\kappa^{2}\right)$.

By using the similar approach as in the exact analysis of outage probability, we can get the results as stated in the following theorem.

**Theorem** **3** (Outage probability—Asymptotic form)**.** *The asymptotic outage probability and achievable throughput for node $S_{1}$ of the proposed half-duplex bidirectional wireless sensor network using time-switching energy harvesting strategy over a Rician fading channel can be provided as*

(22) $$P_{out\_1}^{\infty }=1-\zeta_{1}e^{-2K}\sum_{l=0}^{\infty }\sum_{k=0}^{\infty }\sum_{m=0}^{l}\frac{K^{l+k}\zeta_{1}^{k}\zeta_{2}^{m}}{l!m!{\left(k!\right)}^{2}}{\left(a{\tilde{\gamma }}_{th}\right)}^{m}\frac{\Gamma (m+k+1)}{{\left(\zeta_{2}a{\tilde{\gamma }}_{th}+\zeta_{1}\right)}^{m+k+1}}$$

*and*

(23) $$\tau_{1}≜\left(1-P_{out\_1}^{\infty }\right)\frac{R}{2}=\frac{\zeta_{1}Re^{-2K}}{2}\sum_{l=0}^{\infty }\sum_{k=0}^{\infty }\sum_{m=0}^{l}\frac{K^{l+k}\zeta_{1}^{k}\zeta_{2}^{m}}{l!m!{\left(k!\right)}^{2}}{\left(a{\tilde{\gamma }}_{th}\right)}^{m}\frac{\Gamma (m+k+1)}{{\left(\zeta_{2}a{\tilde{\gamma }}_{th}+\zeta_{1}\right)}^{m+k+1}},$$

*where $a=\kappa^{2}+\kappa_{r}^{2}\left(1+\kappa^{2}\right),b=\frac{\left(1+\kappa_{r}^{2}\right)}{P/\sigma_{r}^{2}},c=\frac{\left(1+\kappa^{2}\right)}{\psi P/\sigma_{i}^{2}},\zeta_{i}=\frac{K+1}{\lambda_{i}},{\tilde{\gamma }}_{th}=\frac{\gamma_{th}}{1-a\gamma_{th}}$, and Γ(·) is the complete Gamma function, which is defined by $\Gamma \left(z\right)≜\int_{0}^{\infty }x^{z-1}e^{-x}dx$.*

**Proof** **of** **Theorem** **3.** See Appendix C. ☐

#### 3.4.2. SER Analysis

As $P/N_{0}$ approaches infinity, the symbol-error-rate at node $S_{1}$ becomes

(24) $${SER}_{1}^{\infty }=\frac{\omega \sqrt{\theta }}{2\sqrt{\pi }}\int_{0}^{\infty }\frac{e^{-\theta x}}{\sqrt{x}}F_{\gamma_{1}}^{\infty }\left(x\right)dx,$$

where $F_{\gamma_{1}}^{\infty }\left(x\right)$ is the CDF of $\gamma_{1}$ as $P/N_{0}$ goes to infinity.

The result of our asymptotic SER analysis is presented in Theorem 4.

**Theorem** **4** (SER—Asymptotic form)**.** *The asymptotic symbol error rate for node $S_{1}$ of the proposed half-duplex bidirectional wireless sensor network using a time-switching energy harvesting strategy over a Rician fading channel can be provided as the following:*

- *If $\xi_{1}=\xi_{2}$:*
  (25) $$\begin{array}{c} {SER}_{1}^{\infty }=\frac{\omega }{2}\\ -\frac{\omega \sqrt{\theta }}{2\sqrt{\pi }}\zeta_{1}e^{-2K}\sum_{l=0}^{\infty }\sum_{k=0}^{\infty }\sum_{m=0}^{l}\sum_{v=0}^{k+1}\left(\begin{array}{c} k+1\\ v \end{array}\right)\frac{K^{l+k}\zeta_{1}^{k}\zeta_{2}^{m}a^{m+v}{\left(-1\right)}^{v}\Gamma \left(m+k+1\right)}{l!m!{\left(k!\right)}^{2}\zeta_{1}^{m+1}\theta^{m+v+\frac{1}{2}}}\gamma \left(m+v+\frac{1}{2},\frac{\theta }{a}\right). \end{array}$$
- *If $\xi_{1}\neq \xi_{2}$:*
  (26) $$\begin{array}{c} SER_{1}^{\infty }=\frac{\omega }{2}-\frac{\omega \sqrt{\theta }}{2\sqrt{\pi }}\zeta_{1}e^{-2K}\sum_{l=0}^{\infty }\sum_{k=0}^{\infty }\sum_{m=0}^{l}\sum_{v=0}^{k+1}\sum_{p=0}^{\infty }{\left(m+k+1\right)}_{p}{\left[(\xi_{1}-\xi_{2})\right]}^{p}\left(\begin{array}{c} k+1\\ v \end{array}\right)\\ \times \frac{K^{l+k}\zeta_{2}^{m}a^{m+v+p}{\left(-1\right)}^{v}\Gamma \left(m+k+1\right)}{l!m!{\left(k!\right)}^{2}p!\zeta_{1}^{m+p+1}\theta^{m+v+p+\frac{1}{2}}}\gamma \left(m+v+p+\frac{1}{2},\frac{\theta }{a}\right), \end{array}$$

*where $a=\kappa^{2}+\kappa_{r}^{2}\left(1+\kappa^{2}\right),b=\frac{\left(1+\kappa_{r}^{2}\right)}{P/\sigma_{r}^{2}},c=\frac{\left(1+\kappa^{2}\right)}{\psi P/\sigma_{i}^{2}},\zeta_{1}=\frac{K+1}{\lambda_{1}},{\tilde{\gamma }}_{th}=\frac{\gamma_{th}}{1-a\gamma_{th}}$, Γ(·) is the complete Gamma function, γ(·,·) is the incomplete Gamma function, and ${\left(\cdot \right)}_{p}$ is the Pochhammer symbol.*

**Proof** **of** **Theorem** **4.** See Appendix D. ☐

### 3.5. Optimal Time-Switching Factor

It is not difficult to learn that there is always a trade-off between the amount of energy used for transmission and the duration of the transmission in the considered protocol. Specifically, if more time is allocated for energy harvesting, a higher available transmission power can be obtained, which may lead to a higher throughput. However, at the same time, less time resources are left for signal transmission, which may lead to the decrease of the transmission rate. Hence, there exists an optimal time-switching factor $\alpha^{*}$ that provides the best throughput performance.

Given the throughput expression obtained in Equation (16), the optimal time switching can be obtained by solving Equation $\frac{d\tau_{i}\left(\alpha \right)}{d\alpha }=0$. However, due to the complicated infinite series and Bessel function in each throughput expression, this optimization problem can hardly admit a closed-form solution.

Here, we apply an iterative algorithm to solve this problem numerically. In particular, the Golden section search algorithm, which has been used in many global optimization problems in communications (for example, in [35]), is chosen for this work. For a detailed algorithm as well as its related theory, please refer to [36].

## 4. Numerical Results and Discussion

For the purpose of validation, the correctness of the derived outage probability and SER expressions as well as investigation of the effect of various parameters on the system performance, a set of Monte Carlo simulations are conducted and presented in this section. For each simulation, we first provide the graphs of the outage probability and throughput obtained by the analytical formulas. Secondly, we plot the same outage probability and throughput curves that result from Monte Carlo simulation. To do this, we generate $10^{5}$ random samples of each channel gain, which are Rician distributed. Using these random samples, the SNR at destination node $S_{1}$ is calculated and compared with the threshold value *γ*. The outage probability occurs if this SNR falls below the threshold. By taking the number of cases that SNR<γ divided by the number of samples, we can estimate the outage probability and then the throughput of system. The analytical curve and the simulation one should match together to verify the correctness of our analysis.

The hardware impairment parameters are chosen as $\kappa =\kappa_{r}=0.1$. The ideal hardware impairment situation ($\kappa =\kappa_{r}=0$) is also considered as a benchmark performance for simulation. The channel gains are considered as Rician fading with $\lambda_{1}=\lambda_{2}=0.5$ and with the Rician K-factors equal to 3 for both channels. The transmit power are set to the same value $P_{1}=P_{2}=P$ for both two sources, so that the ratio $P/N_{0}$ varies in the range from 0 to 50 dB. The energy harvesting efficiency is set to be 0.7. The source transmission rate is chosen as 1.5 bps/Hz. From the Shannon’s theorem on capacity of the channel, we can calculate the SNR threshold as $\gamma =2^{2R}-1$. All simulation parameters are listed in Table 1.

### 4.1. Effects of Various Parameters on the System Performance

**Figure 3:**
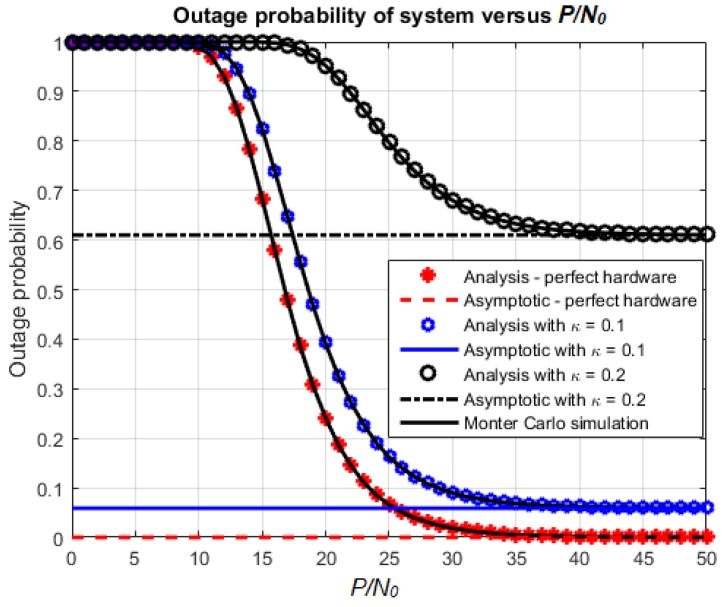
Outage probability versus $P/N_{0}$.

**Figure 4:**
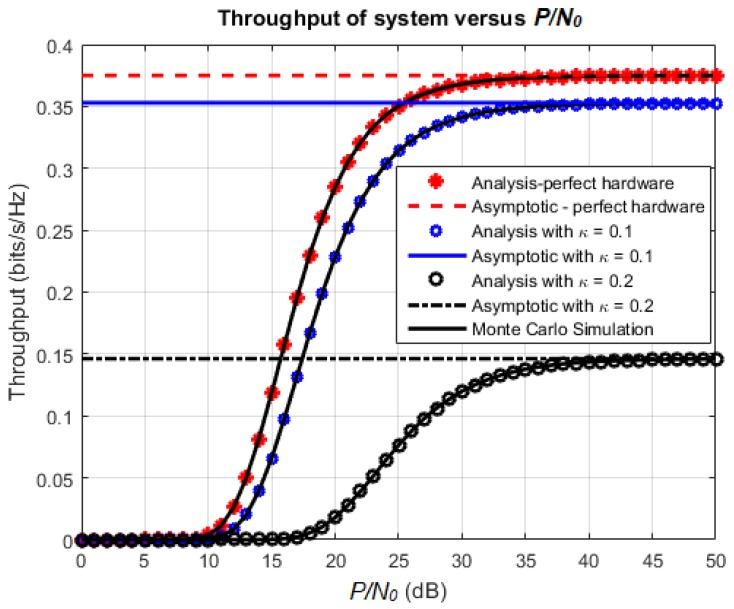
Achievable throughput versus $P/N_{0}$

Figure 3 and Figure 4 show the effect of $P/N_{0}$ on the outage probability and throughput of the proposed system, respectively. For this simulation, the utilized parameter settings are: $\kappa =\kappa_{r}=0.1$ or 0.2,α=0.5 and η=0.7. We choose α=0.5 to consider the case that the duration of energy harvesting and the duration of transmission are balanced. The case κ=0 (no hardware impairment) is also introduced for comparison. The first observation is that the outage probability and throughput obtained from mathematical analysis match with the corresponding Monte Carlo simulations. Regarding the effect of *κ*, the outage probability decreases and the throughput increases as *κ* varies from 0 to 0.2. When $P/N_{0}$ increases, the outage probability and throughput approach the corresponding asymptotic values obtained from analysis. Furthermore, the lower the value of *κ*, the faster the outage probability and throughput converge to their asymptotic values.

**Figure 5:**
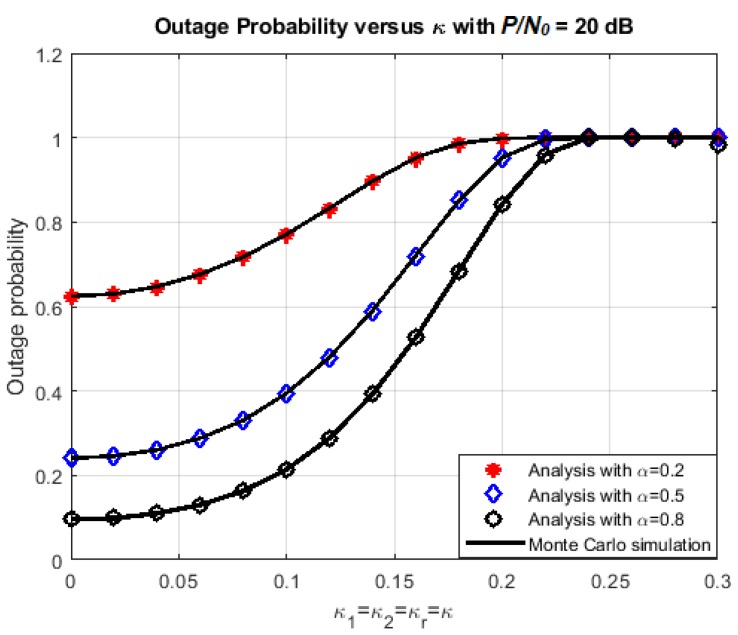
Outage probability versus *κ*.

**Figure 6:**
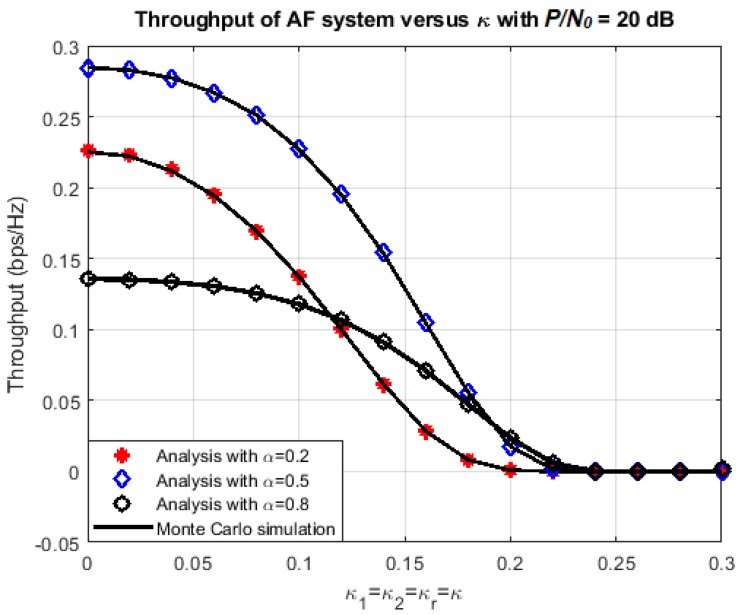
Achievable throughput versus *κ*.

The effect of hardware impairment level on the outage probability and the achievable throughput at each node is presented more thoroughly in Figure 5 and Figure 6. Here, $P/N_{0}$ is set at 20 dB and the transmission rate is fixed at 1.5 bps. Three values of *α* are chosen: 0.2, 0.5, and 0.8, corresponding to three cases: the energy harvesting duration is dominant, there is a balance between energy harvesting and information transmission, and the information transmission duration is dominant.

Again, it is observed that the exact-form expressions of outage probability and throughput obtained by the analysis coincide with the ones that are obtained by Monte Carlo simulations. From the numerical results, it is evident that the achievable throughput decreases and the outage probability increases significantly at each destination node when the impairment level *κ* increases. In addition, the outage probability tends to reduce at higher time-switching factor. This can be explained because the larger value of *α* means more power is used for data transmission. However, this doesn’t mean that the throughput is better for larger *α*. In Figure 6, the throughput performance is improved when *α* increases from 0.2 to 0.5, but then degraded when *α* increases from 0.5 to 0.8.

**Figure 7:**
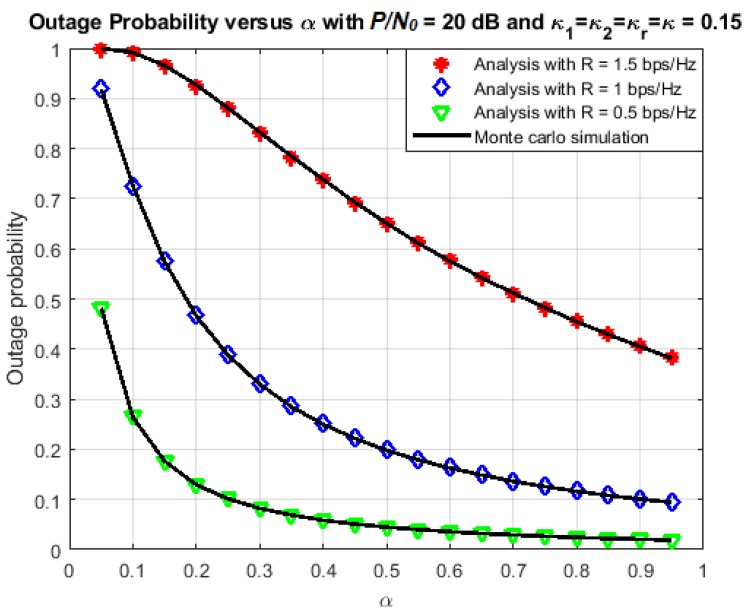
Outage probability versus *α*.

**Figure 8:**
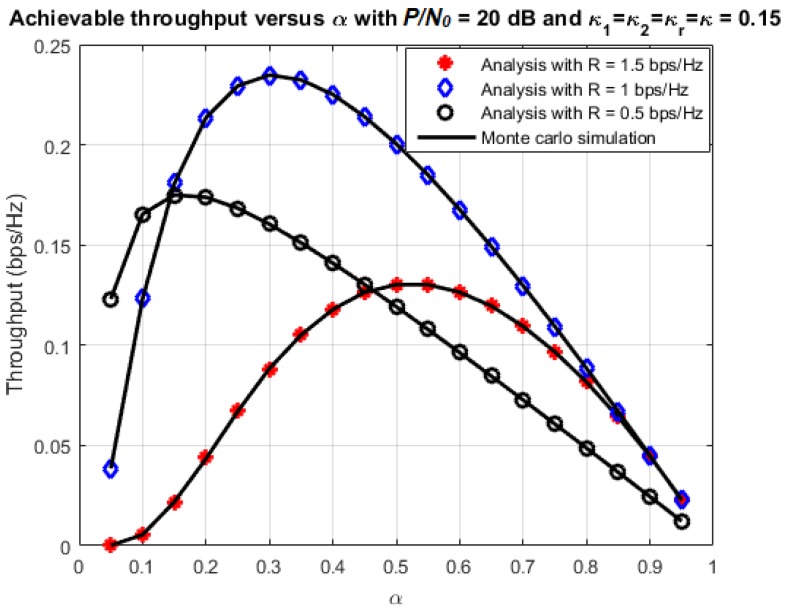
Achievable throughput versus *α*.

Figure 7 and Figure 8 illustrate more clearly the effect of time-switching factor on the outage and throughput performance. In this simulation, the parameters are chosen as η=0.7 and $P/N_{0}=20$ dB (this value is chosen because it is in the middle range of $P/N_{0}$). The transmission rate varies among three values: 0.5 bps/Hz, 1 bps/Hz and 1.5 bps/Hz, while the time-switching factor varies in the range (0,1). The results confirm what we mentioned just above. There should be a unique value of *α* that maximizes the throughput. This is because, when we increase *α* initially, there is more power used for transmission, so the outage probability is reduced and the throughput increases correspondingly. However, when *α* keeps increasing, the duration of transmission is also reduced, hence, less data is transmitted during a given time interval. As a result, the throughput performance becomes worse.

### 4.2. Effect of Various Parameters on SER

**Figure 9:**
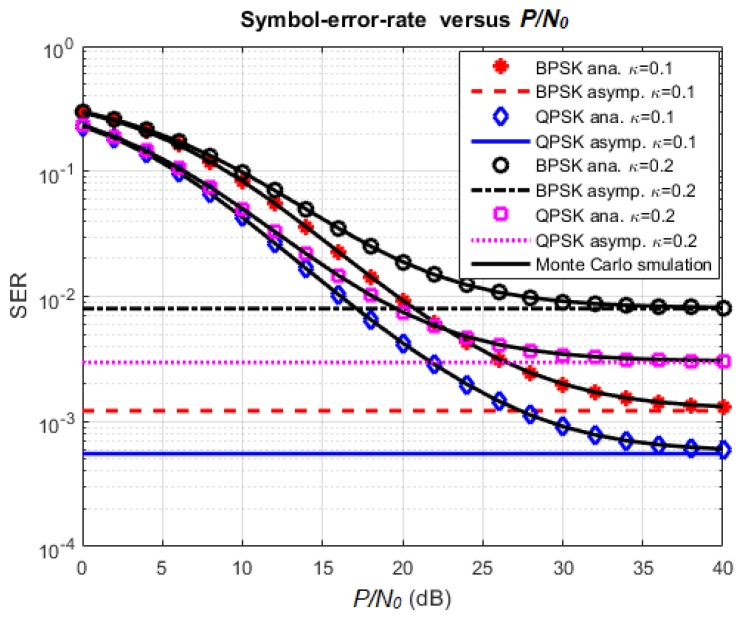
Effect of *κ* and modulation scheme on SER.

The purpose of the following simulations is to confirm the correctness of the SER formulas provided in the analysis. First, Figure 9 presents the effect of the hardware impairment level on the SER performance. In this simulation, the time-switching factor is chosen as α=0.5 with the same reason as in Section 4.1, the transmission rate is fixed at 1 bps/Hz, and the ratio $P/N_{0}$ varies in the range from 0 dB to 40 dB. From the results, it is showed that SER decreases to the asymptotic value when the ratio $P_{s}/N_{0}$ increases. The Monte Carlo simulation curves overlap with the corresponding analysis curves. This confirms the validity of our analysis. When the hardware impairment level goes higher, the SER also has a larger value, as expected. Furthermore, the SER performance of the QPSK scheme is better than the one of the BPSK scheme in the same simulation condition. This can be explained because the QPSK modulation scheme can transmit two bits in one symbol while the BPSK scheme can only transmit one bit per symbol. Hence, if we have the same constraint on the transmission rate for both methods, the required SNR for maintaining good communication would be smaller for the QPSK method. As a result, the outage probability and the SER for QPSK modulation would be smaller than the ones of BPSK.

**Figure 10:**
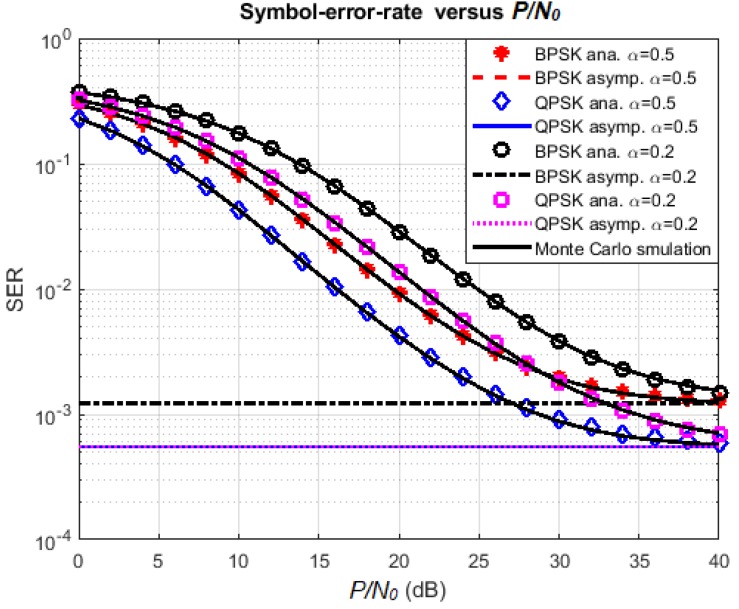
Effect of *α* and modulation scheme on SER.

In a similar way, the influence of the time-switching factor on the symbol-error-rate at the destination node is illustrated in Figure 10. The simulation parameters are κ=0.1,R=1 bps/Hz, and $P/N_{0}$ varies from 0 dB to 40 dB. Again, the simulation curves match perfectly with the corresponding analysis curves. The SER tends to approach its asymptotic value, and QPSK modulation still provides the better SER performance than BPSK for both values of *α*. Note that, for this simulation, the asymptotic values do not depend on the time-switching factor *α*. However, *α* surely has an effect on the immediate value of SER. In fact, the SER performance should be better with the value of *α* that is in the middle of its range. For example, in Figure 10, the SER value for α=0.5 is less than the one with α=0.2. The explanation is the same as the case of Figure 8.

### 4.3. Optimal Time-Switching Factor

**Figure 11:**
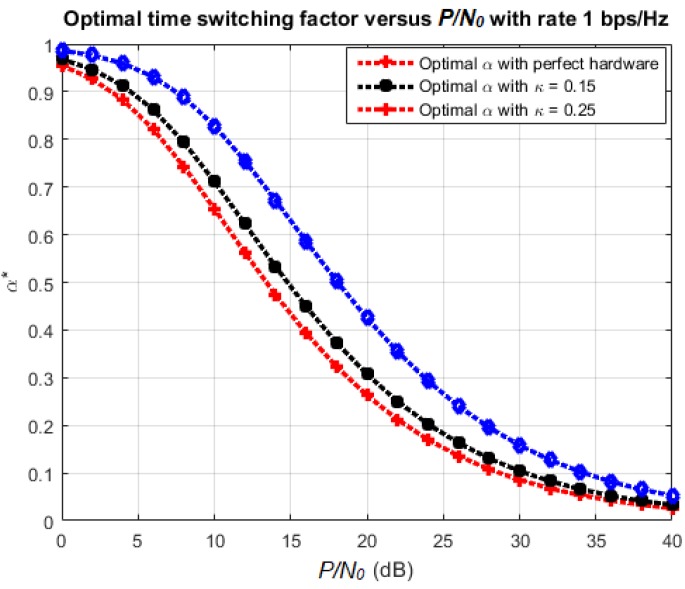
Effect of *α* and modulation scheme on SER.

As mentioned in Section 3, the optimal time-switching factor to maximize the achievable throughput of the considered system can be found numerically by using an iterative algorithm such as the golden section search method [36]. Figure 11 plots the optimal value $\alpha^{*}$ for various values of the ratio $P/N_{0}$ at different hardware impairment levels.

It can be observed that the optimal time-switching factor decreases as the ratio $P/N_{0}$ increases. This is because, for large $P/N_{0}$, the outage probability tends to reduce, so it is not necessary to use a large amount of energy to transmit data. Reversely, we need to spend more time resources to increase the throughput of the system.

On the other hand, we can learn from this simulation that the optimal *α* does not change much for different hardware impairment levels. Especially, for small *κ*, the value of $\alpha^{*}$ is nearly the same as the one for a perfect hardware case.

## 5. Conclusions

Recent development in wireless sensor networks have led to an exponential growth of the energy demand for operating the networks, which raises a question about how to efficiently use the available energy in the wireless environment. This paper rigorously analyzes the performance of a half-duplex AF bidirectional sensor network in which the relay node is equipped with time-switching-based energy harvesting protocol. The channel considered in this paper is a Rician fading channel. We also take into account the hardware impairment at source and relay nodes. We derive both exact and asymptotic forms of the outage probability, achievable throughput, as well as the symbol-error-rate at each destination node. The analysis results are validated by Monte Carlo simulation. From the results of this work, we can gain an insightful understanding of the effect of various parameters on the system performance. Furthermore, the optimal time-switching factor (i.e., the best energy harvesting strategy) is also founded by numerical algorithms.

## Figures and Tables

**Table 1:** Simulation parameters.

Symbol	Parameter Names	Values
η	Energy harvesting efficiency	0.7
$\lambda_{1}$	Mean of $|g_{1}{|}^{2}$	0.5
$\lambda_{2}$	Mean of $|g_{2}{|}^{2}$	0.5
*K*	Rician K-factor	3
$P/N_{0}$	Source-power-to-noise ratio	0–50 dB
$\kappa =\kappa_{r}$	Hardware impairment levels	0, 0.15, 0.25
*R*	Source transmission rate	1.5 bps/Hz

## Abbreviations

The following abbreviations are used in this manuscript:

AF	amplify-and-forward
AWGN	additive white Gaussian noise
BPSK	binary phase shift keying
CDF	cumulative distribution function
EH	energy harvesting
I/Q	In-phase/Quadrature
LOS	line-of-sight
PDF	probability density function
PS	power splitting
QPSK	quadrature phase shift keying
RF	radio frequency
RV	random variable
SER	symbol-error-rate
SNR	signal-to-noise ratio
SWIPT	simultaneous wireless information and power transfer
TS	time switching
TWRN	two-way relay network
WSN	wireless sensor network

## Appendix A. Proof of Theorem 1

In this section, the detailed proof of Theorem 1 is provided. Without loss of generality, we can consider the node $S_{1}$ only. From Equation (14), we can fix $X_{1}$ at some value $x_{1}$ and compute the probability in Equation (14) in terms of $x_{1}$. Then, we take the average of the obtained function with respect to the distribution of $X_{1}$ to get the outage probability. Using this approach, Equation (14) can be rewritten as

(A1) $$P_{out\_1}=\int_{0}^{\infty }F_{X_{2}}\left(\frac{\gamma_{th}x_{1}^{2}a+\gamma_{th}x_{1}b+\gamma_{th}c}{x_{1}-\gamma_{th}x_{1}a}\right)f_{X_{1}}\left(x_{1}\right)dx_{1}.$$

Let us denote ${\tilde{\gamma }}_{th}≜\frac{\gamma_{th}}{1-a\gamma_{th}}$. Then, we have $\frac{\gamma_{th}x_{1}^{2}a+\gamma_{th}x_{1}b+\gamma_{th}c}{x_{1}-\gamma_{th}x_{1}a}={\tilde{\gamma }}_{th}\left(x_{1}a+\frac{c}{x_{1}}+b\right)$. Now, Equation (A1) can be rewritten as

(A2) $$\begin{array}{c} P_{out\_1}=\\ =1-\int_{0}^{\infty }\sum_{l=0}^{\infty }\sum_{m=0}^{l}\frac{e^{-K}K^{l}{\left(\zeta_{2}{\tilde{\gamma }}_{th}\right)}^{m}}{l!m!}{\left(x_{1}a+\frac{c}{x_{1}}+b_{1}\right)}^{m}e^{-\zeta_{2}{\tilde{\gamma }}_{th}\left(ax_{1}+\frac{c}{x_{1}}+b\right)}\zeta_{1}e^{-K}\sum_{k=0}^{\infty }\frac{{\left(\zeta_{1}Kx_{1}\right)}^{k}e^{-\zeta_{1}x_{1}}}{{\left(k!\right)}^{2}}dx_{1}\\ =1-\zeta_{1}e^{-2K}\int_{0}^{\infty }{\sum_{l=0}^{\infty }\sum_{k=0}^{\infty }\sum_{m=0}^{l}\frac{K^{l+k}\zeta_{2}^{m}{\tilde{\gamma }}_{th}^{m}}{l!m!{\left(k!\right)}^{2}}\left(x_{1}a+\frac{c}{x_{1}}+b\right)}^{m}e^{-\zeta_{2}{\tilde{\gamma }}_{th}\left(x_{1}a+\frac{c}{x_{1}}+b\right)}{\left(\zeta_{1}x_{1}\right)}^{k}e^{-\zeta_{1}x_{1}}dx_{1}. \end{array}$$

By applying the formula ${\left(x+y\right)}^{m}=\sum_{n=0}^{m}\left(\begin{array}{c} m\\ n \end{array}\right)x^{m-n}y^{n}$ and letting $\xi_{12}≜\zeta_{1}e^{-2K}e^{-b\zeta_{2}{\tilde{\gamma }}_{th}}$ , we obtain:

(A3) $$\begin{array}{cc} P_{out\_1} & =1-\xi_{12}\int_{0}^{\infty }\sum_{l=0}^{\infty }\sum_{k=0}^{\infty }\sum_{m=0}^{l}\frac{K^{l+k}\zeta_{2}^{m}{\tilde{\gamma }}_{th}^{m}}{l!m!{\left(k!\right)}^{2}}\sum_{n=0}^{m}\left(\begin{array}{c} m\\ n \end{array}\right){\left(x_{1}a\right)}^{m-n}{\left(\frac{c}{x_{1}}+b\right)}^{n}e^{-\zeta_{2}{\tilde{\gamma }}_{th}\left(x_{1}a+\frac{c}{x_{1}}\right)}{\left(\zeta_{1}x_{1}\right)}^{k}e^{-\zeta_{1}x_{1}}dx_{1}\\ & =1-\xi_{12}\int_{0}^{\infty }\sum_{l=0}^{\infty }\sum_{k=0}^{\infty }\sum_{m=0}^{l}\sum_{n=0}^{m}\frac{K^{l+k}\zeta_{1}^{k}\zeta_{2}^{m}{\tilde{\gamma }}_{th}^{m}a^{m-n}}{l!n!\left(m-n\right)!{\left(k!\right)}^{2}}{x_{1}}^{m-n+k}\sum_{p=0}^{n}\left(\begin{array}{c} n\\ p \end{array}\right){\left(\frac{c}{x_{1}}\right)}^{n-p}b^{p}e^{-\zeta_{2}{\tilde{\gamma }}_{th}\left(x_{1}a+\frac{c}{x_{1}}\right)}e^{-\zeta_{1}x_{1}}dx_{1}\\ & =1-\xi_{12}\int_{0}^{\infty }\sum_{l=0}^{\infty }\sum_{k=0}^{\infty }\sum_{m=0}^{l}\sum_{n=0}^{m}\sum_{p=0}^{n}\frac{K^{l+k}\zeta_{1}^{k}\zeta_{2}^{m}{\tilde{\gamma }}_{th}^{m}a^{m-n}b^{p}c^{n-p}}{l!p!\left(n-p\right)!\left(m-n\right)!{\left(k!\right)}^{2}}{x_{1}}^{m-2n+k+p}e^{-\zeta_{2}{\tilde{\gamma }}_{th}\left(x_{1}a+\frac{c}{x_{1}}\right)}e^{-\zeta_{1}x_{1}}dx_{1}\\ & =1-\xi_{12}\sum_{l=0}^{\infty }\sum_{k=0}^{\infty }\sum_{m=0}^{l}\sum_{n=0}^{m}\sum_{p=0}^{n}\frac{K^{l+k}\zeta_{1}^{k}\zeta_{2}^{m}{\tilde{\gamma }}_{th}^{m}a^{m-n}b^{p}c^{n-p}}{l!p!\left(n-p\right)!\left(m-n\right)!{\left(k!\right)}^{2}}\int_{0}^{\infty }{x_{1}}^{m-2n+k+p}e^{-\left(\zeta_{1}+a\zeta_{2}{\tilde{\gamma }}_{th}\right)x_{1}-\zeta_{2}{\tilde{\gamma }}_{th}\frac{c}{x_{1}}}dx_{1}. \end{array}$$

Now, by letting $\zeta_{12}≜\zeta_{1}+a\zeta_{2}{\tilde{\gamma }}_{th}$ and using Equation (3.471.9) in [32], we obtain the outage probability:

(A4) $$\begin{array}{cc} P_{out\_1} & =1-\xi_{12}\sum_{l=0}^{\infty }\sum_{k=0}^{\infty }\sum_{m=0}^{l}\sum_{n=0}^{m}\sum_{p=0}^{n}\frac{K^{l+k}\zeta_{1}^{k}\zeta_{2}^{m}{\tilde{\gamma }}_{th}^{m}a^{m-n}b^{p}c^{n-p}}{l!p!\left(n-p\right)!\left(m-n\right)!{\left(k!\right)}^{2}}2{\left(\frac{\zeta_{2}{\tilde{\gamma }}_{th}c}{\zeta_{12}}\right)}^{\frac{m-2n+p+k+1}{2}}K_{m-2n+p+k+1}\left(2\sqrt{\zeta_{12}\zeta_{2}{\tilde{\gamma }}_{th}c}\right)\\ & =1-2\xi_{12}\sum_{l=0}^{\infty }\sum_{k=0}^{\infty }\sum_{m=0}^{l}\sum_{n=0}^{m}\sum_{p=0}^{n}\frac{K^{l+k}\zeta_{1}^{k}{\left(\zeta_{2}{\tilde{\gamma }}_{th}\right)}^{\frac{3m-2n+p+k+1}{2}}a^{m-n}b^{p}c^{\frac{m-p+k+1}{2}}}{l!p!\left(n-p\right)!\left(m-n\right)!{\left(k!\right)}^{2}\zeta_{12}^{\frac{m-2n+p+k+1}{2}}}K_{m-2n+p+k+1}\left(2\sqrt{\zeta_{12}\zeta_{2}{\tilde{\gamma }}_{th}c}\right), \end{array}$$

where $K_{v}\left(\cdot \right)$ is the $\nu^{th}$-order modified Bessel function of the second kind.

## Appendix B. Proof of Theorem 2

Again, without loss of generality, we can only consider the case i=1 (the SER at node 1). Obviously, the CDF of the SNR at node $S_{1}$, denoted by $F_{\gamma_{1}}\left(x\right)$, is the outage probability at $S_{1}$ when the threshold SNR is equal to *x*. Thus, it can be written as

(A5) $$F_{\gamma_{1}}\left(x\right)=\mathrm{Pr}\left(\frac{X_{1}X_{2}}{X_{1}X_{2}a+X_{1}^{2}a+X_{1}b+c}<x\right)=\left\{\begin{array}{c} \mathrm{Pr}\left(X_{2}<\frac{xX_{1}^{2}a+xX_{1}b+xc}{X_{1}-xX_{1}a}\right),\;\mathrm{if}\;x⩽\frac{1}{a},\\ 1,\;\;\;\;\;\;\;\;\;\;\;\;\mathrm{if}\;x>\frac{1}{a}. \end{array}\right.$$

Then, we can substitute Equation (A5) into Equation (19) to get

(A6) $$\begin{array}{c} {SER}_{1}=\frac{\omega \sqrt{\theta }}{2\sqrt{\pi }}\int_{0}^{\infty }\frac{e^{-\theta x}}{\sqrt{x}}F_{\gamma_{1}}\left(x\right)dx=\frac{\omega \sqrt{\theta }}{2\sqrt{\pi }}\int_{0}^{1/a}\frac{e^{-\theta x}}{\sqrt{x}}F_{\gamma_{1}}\left(x\right)dx+\frac{\omega \sqrt{\theta }}{2\sqrt{\pi }}\int_{1/a}^{\infty }\frac{e^{-\theta x}}{\sqrt{x}}dx. \end{array}$$

By using the results from the previous version, the CDF $F_{\gamma_{1}}\left(x\right)$ for x≤1/a is given by

(A7) $$\begin{array}{c} F_{\gamma_{1}}\left(x\right)=1-2\zeta_{1}e^{-2K}e^{-b\zeta_{2}{\tilde{\gamma }}_{th}}\sum_{l=0}^{\infty }\sum_{k=0}^{\infty }\sum_{m=0}^{l}\sum_{n=0}^{m}\sum_{p=0}^{n}\frac{K^{l+k}\zeta_{1}^{k}{\left(\zeta_{2}{\tilde{\gamma }}_{th}\right)}^{\frac{3m-2n+p+k+1}{2}}a^{m-n}b^{p}c^{\frac{m-p+k+1}{2}}}{l!p!\left(n-p\right)!\left(m-n\right)!{\left(k!\right)}^{2}{\left(\zeta_{1}+a\zeta_{2}{\tilde{\gamma }}_{th}\right)}^{\frac{m-2n+p+k+1}{2}}}\\ \times K_{m-2n+p+k+1}\left(2\sqrt{\left(\zeta_{1}+a\zeta_{2}{\tilde{\gamma }}_{th}\right)\zeta_{2}{\tilde{\gamma }}_{th}c}\right). \end{array}$$

Let us denote $\Xi \left(x\right)≜1-F_{\gamma_{1}}\left(x\right)$. Then, Equation (A6) can be rewritten as

(A8) $$\begin{array}{cc} {SER}_{1} & =\frac{\omega \sqrt{\theta }}{2\sqrt{\pi }}\int_{0}^{1/a}\frac{e^{-\theta x}}{\sqrt{x}}\left\{1-\Xi (x)\right\}dx+\frac{\omega \sqrt{\theta }}{2\sqrt{\pi }}\int_{1/a_{1}}^{\infty }\frac{e^{-\theta x}}{\sqrt{x}}dx=\frac{\omega \sqrt{\theta }}{2\sqrt{\pi }}\int_{0}^{\infty }\frac{e^{-\theta x}}{\sqrt{x}}dx-\frac{\omega \sqrt{\theta }}{2\sqrt{\pi }}\int_{0}^{1/a}\frac{e^{-\theta x}}{\sqrt{x}}\Xi \left(x\right)dx\\ & =\frac{\omega }{2}-\frac{\omega \sqrt{\theta }}{2\sqrt{\pi }}\int_{0}^{1/a}\frac{e^{-\theta x}}{\sqrt{x}}\Xi \left(x\right)dx. \end{array}$$

Then, Equation (19) can be obtained by substituting $\Xi \left(x\right)≜1-F_{\gamma_{1}}\left(x\right)$ and Equation (A7) into Equation (18).

## Appendix C. Proof of Theorem 3

The PDF of the random variable $X_{i}$ is given by $f_{X_{i}}\left(x\right)=\zeta_{i}e^{-K}\sum_{l=0}^{\infty }\frac{{\left(\zeta_{i}K\right)}^{l}}{{\left(l!\right)}^{2}}x^{l}e^{-\zeta_{i}x}$. Hence, the asymptotic outage probability at node $S_{1}$ can be written as

$$\begin{array}{cc} P_{out\_1}^{\infty } & =\mathrm{Pr}\left(\gamma_{1}^{\infty }<\gamma_{th}\right)=\mathrm{Pr}\left(\frac{X_{2}}{X_{1}+X_{2}}<a\gamma_{th}\right)=\mathrm{Pr}\left(X_{2}<\frac{a\gamma_{th}X_{1}}{1-a\gamma_{th}}\right)\\ & =\int_{0}^{\infty }F_{X_{2}}\left(\frac{a\gamma_{th}x_{1}}{1-a\gamma_{th}}\right)f_{X_{1}}\left(x_{1}\right)dx_{1}\\ & =1-\int_{0}^{\infty }e^{-K}\sum_{l=0}^{\infty }\sum_{m=0}^{l}\frac{K^{l}\zeta_{2}^{m}}{l!m!}{\left(a{\tilde{\gamma }}_{th}x_{1}\right)}^{m}e^{-\zeta_{2}a{\tilde{\gamma }}_{th}x_{1}}\zeta_{1}e^{-K}\sum_{k=0}^{\infty }\frac{{\left(\zeta_{1}K\right)}^{k}}{{\left(k!\right)}^{2}}x_{1}^{k}e^{-\zeta_{1}x_{1}}dx_{1}\\ & =1-\zeta_{1}e^{-2K}\int_{0}^{\infty }\sum_{l=0}^{\infty }\sum_{k=0}^{\infty }\sum_{m=0}^{l}\frac{K^{l+k}\zeta_{1}^{k}\zeta_{2}^{m}}{l!m!{\left(k!\right)}^{2}}{\left(a{\tilde{\gamma }}_{th}x_{1}\right)}^{m}e^{-\zeta_{2}a{\tilde{\gamma }}_{th}x_{1}}x_{1}^{k}e^{-\zeta_{1}x_{1}}dx_{1}\\ & =1-\zeta_{1}e^{-2K}\sum_{l=0}^{\infty }\sum_{k=0}^{\infty }\sum_{m=0}^{l}\frac{K^{l+k}\zeta_{1}^{k}\zeta_{2}^{m}}{l!m!{\left(k!\right)}^{2}}{\left(a{\tilde{\gamma }}_{th}\right)}^{m}\int_{0}^{\infty }x_{1}^{m+k}.e^{-\left(\zeta_{2}a{\tilde{\gamma }}_{th}+\zeta_{1}\right)x_{1}}dx_{1}. \end{array}$$

Using the Formula (3.381.4) in [32], we obtain the asymptotic formula:

(A9) $$\begin{array}{cc} P_{out\_1}^{\infty } & =1-\zeta_{1}e^{-2K}\sum_{l=0}^{\infty }\sum_{k=0}^{\infty }\sum_{m=0}^{l}\frac{K^{l+k}\zeta_{1}^{k}\zeta_{2}^{m}}{l!m!{\left(k!\right)}^{2}}{\left(a{\tilde{\gamma }}_{th}\right)}^{m}\frac{\Gamma (m+k+1)}{{\left(\zeta_{2}a{\tilde{\gamma }}_{th}+\zeta_{1}\right)}^{m+k+1}}, \end{array}$$

where Γ(·) is the complete gamma function.

The asymptotic throughput can be obtained by substituting Equation (A9) into the definitive formula of the throughput.

## Appendix D. Proof of Theorem 4

The CDF of $\gamma_{1}$ as high SNR regime can be written as

(A10) $$F_{\gamma_{1}^{\infty }}\left(x\right)=\mathrm{Pr}\left(\gamma_{1}^{\infty }<x\right)=\mathrm{Pr}\left(\frac{X_{2}}{aX_{2}+aX_{1}}<x\right)=\left\{\begin{array}{c} \mathrm{Pr}\left(X_{2}<\frac{axX_{1}}{1-ax}\right),\;\mathrm{if}\;x⩽\frac{1}{a},\\ 1,\;\;\;\;\;\;\;\;\mathrm{if}\;x>\frac{1}{a}. \end{array}\right.$$

Now, we can rewrite the formula of SER by substituting Equation (A10) into Equation (24)

(A11) $$SER_{1}^{\infty }=\frac{\omega \sqrt{\theta }}{2\sqrt{\pi }}\int_{0}^{\infty }\frac{e^{-\theta x}}{\sqrt{x}}F_{\gamma_{1}}^{\infty }\left(x\right)dx=\frac{\omega \sqrt{\theta }}{2\sqrt{\pi }}\int_{0}^{1/a}\frac{e^{-\theta x}}{\sqrt{x}}F_{\gamma_{1}}^{\infty }\left(x\right)dx+\frac{\omega \sqrt{\theta }}{2\sqrt{\pi }}\int_{1/a}^{\infty }\frac{e^{-\theta x}}{\sqrt{x}}dx.$$

For $x\leq \frac{1}{a}$, we can apply the result of Theorem 3 with ${\tilde{\gamma }}_{th}\left(x\right)=\frac{x}{1-ax}$ to get

(A12) $$\begin{array}{cc} F_{\gamma_{1}}^{\infty }\left(x\right) & =\mathrm{Pr}\left(\gamma_{1}^{\infty }<x\right)=1-\zeta_{1}e^{-2K}\sum_{l=0}^{\infty }\sum_{k=0}^{\infty }\sum_{m=0}^{l}\frac{K^{l+k}\zeta_{1}^{k}\zeta_{2}^{m}}{l!m!{\left(k!\right)}^{2}}{\left(a{\tilde{\gamma }}_{th}\left(x\right)\right)}^{m}\frac{\Gamma (m+k+1)}{{\left[\zeta_{2}a{\tilde{\gamma }}_{th}\left(x\right)+\zeta_{1}\right]}^{m+k+1}}\\ & =1-\zeta_{1}e^{-2K}\sum_{l=0}^{\infty }\sum_{k=0}^{\infty }\sum_{m=0}^{l}\frac{K^{l+k}\zeta_{1}^{k}\zeta_{2}^{m}a^{m}}{l!m!{\left(k!\right)}^{2}}.\frac{x^{m}{\left(1-ax\right)}^{k+1}\Gamma \left(m+k+1\right)}{{\left[\left(\zeta_{2}-\zeta_{1}\right)ax+\zeta_{1}\right]}^{m+k+1}}\\ & =1-\zeta_{1}e^{-2K}\sum_{l=0}^{\infty }\sum_{k=0}^{\infty }\sum_{m=0}^{l}\sum_{v=0}^{k+1}\left(\begin{array}{c} k+1\\ v \end{array}\right)\frac{K^{l+k}\zeta_{1}^{k}\zeta_{2}^{m}a^{m+v}{\left(-1\right)}^{v}x^{m+v}\Gamma \left(m+k+1\right)}{l!m!{\left(k!\right)}^{2}{\left[\left(\zeta_{2}-\zeta_{1}\right)ax+\zeta_{1}\right]}^{m+k+1}}\\ & =1-\zeta_{1}e^{-2K}\sum_{l=0}^{\infty }\sum_{k=0}^{\infty }\sum_{m=0}^{l}\sum_{v=0}^{k+1}\left(\begin{array}{c} k+1\\ v \end{array}\right)\frac{K^{l+k}\zeta_{2}^{m}a^{m+v}{\left(-1\right)}^{v}x^{m+v}\Gamma \left(m+k+1\right)}{l!m!{\left(k!\right)}^{2}\zeta_{1}^{m+1}{\left[1+\frac{(\zeta_{2}-\zeta_{1})ax}{\zeta_{1}}\right]}^{m+k+1}}. \end{array}$$

The aymptotic SER is now obtained by substituting Equation (A12) into Equation (A11):

(A13) $$\begin{array}{c} SER_{1}^{\infty }=\frac{\omega \sqrt{\theta }}{2\sqrt{\pi }}\int_{0}^{\infty }\frac{e^{-\theta x}}{\sqrt{x}}dx\\ -\frac{\omega \sqrt{\theta }}{2\sqrt{\pi }}\zeta_{1}e^{-2K}\sum_{l=0}^{\infty }\sum_{k=0}^{\infty }\sum_{m=0}^{l}\sum_{v=0}^{k+1}\left(\begin{array}{c} k+1\\ v \end{array}\right)\frac{K^{l+k}\zeta_{1}^{k}\zeta_{2}^{m}a^{v-k-1}{\left(-1\right)}^{v}\Gamma \left(m+k+1\right)}{l!m!{\left(k!\right)}^{2}{\left(\zeta_{2}-\zeta_{1}\right)}^{m+k+1}}\int_{0}^{\infty }\frac{x^{m+v-\frac{1}{2}}e^{-\theta x}dx}{{\left[x+\frac{\zeta_{1}}{(\zeta_{2}-\zeta_{1})a}\right]}^{m+k+1}}\\ =\frac{\omega }{2}-\frac{\omega \sqrt{\theta }}{2\sqrt{\pi }}\zeta_{1}e^{-2K}\sum_{l=0}^{\infty }\sum_{k=0}^{\infty }\sum_{m=0}^{l}\sum_{v=0}^{k+1}\left(\begin{array}{c} k+1\\ v \end{array}\right)\frac{K^{l+k}\zeta_{1}^{k}\zeta_{2}^{m}a^{v-k-1}{\left(-1\right)}^{v}\Gamma \left(m+k+1\right)}{l!m!{\left(k!\right)}^{2}{\left(\zeta_{2}-\zeta_{1}\right)}^{m+k+1}}\int_{0}^{1/a}\frac{x^{m+v-\frac{1}{2}}e^{-\theta x}dx}{{\left[x+\frac{\zeta_{1}}{(\zeta_{2}-\zeta_{1})a}\right]}^{m+k+1}}. \end{array}$$

We consider three cases:

**Case 1:** ξ 2 =ξ 1 .

Equation (A13) can be rewritten as

(A14) $$\begin{array}{c} {SER}_{1}^{\infty }=\frac{\omega }{2}-\frac{\omega \sqrt{\theta }}{2\sqrt{\pi }}\zeta_{1}e^{-2K}\sum_{l=0}^{\infty }\sum_{k=0}^{\infty }\sum_{m=0}^{l}\sum_{v=0}^{k+1}\left(\begin{array}{c} k+1\\ v \end{array}\right)\frac{K^{l+k}\zeta_{1}^{k}\zeta_{2}^{m}a^{m+v}{\left(-1\right)}^{v}\Gamma \left(m+k+1\right)}{l!m!{\left(k!\right)}^{2}\zeta_{1}^{m+1}}\int_{0}^{1/a}x^{m+v-\frac{1}{2}}e^{-\theta x}dx\\ =\frac{\omega }{2}-\frac{\omega \sqrt{\theta }}{2\sqrt{\pi }}\zeta_{1}e^{-2K}\sum_{l=0}^{\infty }\sum_{k=0}^{\infty }\sum_{m=0}^{l}\sum_{v=0}^{k+1}\left(\begin{array}{c} k+1\\ v \end{array}\right)\frac{K^{l+k}\zeta_{1}^{k}\zeta_{2}^{m}a^{m+v}{\left(-1\right)}^{v}\Gamma \left(m+k+1\right)}{l!m!{\left(k!\right)}^{2}\zeta_{1}^{m+1}}\theta^{-m-v-\frac{1}{2}}\gamma \left(m+v+\frac{1}{2},\frac{\theta }{a}\right), \end{array}$$

where $\gamma \left(\alpha ,z\right)≜\int_{0}^{z}e^{-t}t^{\alpha -1}dt$ is the incomplete gamma function as defined in Equation (8.350.1) in [32] and the last equality comes from the Formula (3.381.1) in [32].

**Case 2:** ξ 2 <ξ 1 .

For all $x\in [0,\frac{1}{a}]$, we have $x<\frac{1}{a}.\frac{\xi_{1}}{\xi_{1}-\xi_{2}}$. Hence, we can apply the Taylor’s series expansion of $\frac{1}{{\left[1-\frac{(\xi_{1}-\xi_{2})ax}{\xi_{1}}\right]}^{m+k+1}}$ as in [37]:

(A15) $$\frac{1}{{\left[1-\frac{(\xi_{1}-\xi_{2})ax}{\xi_{1}}\right]}^{m+k+1}}=\sum_{p=0}^{\infty }\frac{{\left(m+k+1\right)}_{p}}{p!}{\left[\frac{(\xi_{1}-\xi_{2})ax}{\xi_{1}}\right]}^{p},$$

where ${\left(\cdot \right)}_{p}$ is the Pochhammer symbol, which is defined as

(A16) $${\left(\alpha \right)}_{p}≜\left\{\begin{array}{c} 1,\;\mathrm{if}\;p=0,\\ \alpha (\alpha +1)...(\alpha +p-1),\;\mathrm{if}\;p\neq 0. \end{array}\right.$$

Now, we can obtain the following equation by substituting Equation (A15) into Equation (A13) and applying (3.381.1) in [32]:

(A17) $$\begin{array}{c} SER_{1}^{\infty }=\frac{\omega }{2}\\ -\frac{\omega \sqrt{\theta }}{2\sqrt{\pi }}\zeta_{1}e^{-2K}\sum_{l=0}^{\infty }\sum_{k=0}^{\infty }\sum_{m=0}^{l}\sum_{v=0}^{k+1}\left(\begin{array}{c} k+1\\ v \end{array}\right)\frac{K^{l+k}\zeta_{1}^{k}\zeta_{2}^{m}a^{v-k-1}{\left(-1\right)}^{v}\Gamma \left(m+k+1\right)}{l!m!{\left(k!\right)}^{2}{\left(\zeta_{2}-\zeta_{1}\right)}^{m+k+1}}\int_{0}^{1/a}\frac{x^{m+v-\frac{1}{2}}e^{-\theta x}dx}{{\left[x+\frac{\zeta_{1}}{(\zeta_{2}-\zeta_{1})a}\right]}^{m+k+1}}\\ =\frac{\omega }{2}-\frac{\omega \sqrt{\theta }}{2\sqrt{\pi }}\zeta_{1}e^{-2K}\sum_{l=0}^{\infty }\sum_{k=0}^{\infty }\sum_{m=0}^{l}\sum_{v=0}^{k+1}\left(\begin{array}{c} k+1\\ v \end{array}\right)\frac{K^{l+k}\zeta_{2}^{m}a^{m+v}{\left(-1\right)}^{v}\Gamma \left(m+k+1\right)}{l!m!{\left(k!\right)}^{2}\zeta_{1}^{m+1}}\int_{0}^{1/a}\frac{x^{m+v-\frac{1}{2}}e^{-\theta x}dx}{{\left[1+\frac{(\zeta_{2}-\zeta_{1})ax}{\zeta_{1}}\right]}^{m+k+1}}\\ =\frac{\omega }{2}-\frac{\omega \sqrt{\theta }}{2\sqrt{\pi }}\zeta_{1}e^{-2K}\sum_{l=0}^{\infty }\sum_{k=0}^{\infty }\sum_{m=0}^{l}\sum_{v=0}^{k+1}\left(\begin{array}{c} k+1\\ v \end{array}\right)\frac{K^{l+k}\zeta_{2}^{m}a^{m+v}{\left(-1\right)}^{v}\Gamma \left(m+k+1\right)}{l!m!{\left(k!\right)}^{2}\zeta_{1}^{m+1}}\\ \times \int_{0}^{1/a}x^{m+v-\frac{1}{2}}e^{-\theta x}\sum_{p=0}^{\infty }\frac{{\left(m+k+1\right)}_{p}}{p!}{\left[\frac{(\xi_{1}-\xi_{2})ax}{\xi_{1}}\right]}^{p}dx\\ =\frac{\omega }{2}-\frac{\omega \sqrt{\theta }}{2\sqrt{\pi }}\zeta_{1}e^{-2K}\sum_{l=0}^{\infty }\sum_{k=0}^{\infty }\sum_{m=0}^{l}\sum_{v=0}^{k+1}\sum_{p=0}^{\infty }{\left(m+k+1\right)}_{p}{\left[(\xi_{1}-\xi_{2})\right]}^{p}\left(\begin{array}{c} k+1\\ v \end{array}\right)\frac{K^{l+k}\zeta_{2}^{m}a^{m+v+p}{\left(-1\right)}^{v}\Gamma \left(m+k+1\right)}{l!m!{\left(k!\right)}^{2}p!\zeta_{1}^{m+p+1}}\\ \times \int_{0}^{1/a}x^{m+v+p-\frac{1}{2}}e^{-\theta x}dx\\ =\frac{\omega }{2}-\frac{\omega \sqrt{\theta }}{2\sqrt{\pi }}\zeta_{1}e^{-2K}\sum_{l=0}^{\infty }\sum_{k=0}^{\infty }\sum_{m=0}^{l}\sum_{v=0}^{k+1}\sum_{p=0}^{\infty }{\left(m+k+1\right)}_{p}{\left[(\xi_{1}-\xi_{2})\right]}^{p}\left(\begin{array}{c} k+1\\ v \end{array}\right)\frac{K^{l+k}\zeta_{2}^{m}a^{m+v+p}{\left(-1\right)}^{v}\Gamma \left(m+k+1\right)}{l!m!{\left(k!\right)}^{2}p!\zeta_{1}^{m+p+1}\theta^{m+v+p+\frac{1}{2}}}\\ \times \gamma \left(m+v+p+\frac{1}{2},\frac{\theta }{a}\right). \end{array}$$

**Case 3:** ξ 2 >ξ 1 .

Using the same approach as in Case 2, with the Taylor’s series expansion $\frac{1}{{\left[1+\frac{(\xi_{2}-\xi_{1})ax}{\xi_{1}}\right]}^{m+k+1}}=\sum_{p=0}^{\infty }{\left(-1\right)}^{p}\frac{{\left(m+k+1\right)}_{p}}{p!}{\left[\frac{(\xi_{2}-\xi_{1})ax}{\xi_{1}}\right]}^{p}$, we get the following result:

(A18) $$\begin{array}{c} SER_{1}^{\infty }=\frac{\omega }{2}-\frac{\omega \sqrt{\theta }}{2\sqrt{\pi }}\zeta_{1}e^{-2K}\sum_{l=0}^{\infty }\sum_{k=0}^{\infty }\sum_{m=0}^{l}\sum_{v=0}^{k+1}\sum_{p=0}^{\infty }{\left(m+k+1\right)}_{p}{\left[(\xi_{1}-\xi_{2})\right]}^{p}\left(\begin{array}{c} k+1\\ v \end{array}\right)\\ \times \frac{K^{l+k}\zeta_{2}^{m}a^{m+v+p}{\left(-1\right)}^{v}\Gamma \left(m+k+1\right)}{l!m!{\left(k!\right)}^{2}p!\zeta_{1}^{m+p+1}\theta^{m+v+p+\frac{1}{2}}}\gamma \left(m+v+p+\frac{1}{2},\frac{\theta }{a}\right). \end{array}$$

## References

[1] De Rango F., Lonetti P., Marano S. (2008). MEA-DSR: A multipath energy-aware routing protocol for wireless Ad Hoc Networks. IFIP Int. Fed. Inf. Process..

[2] Nguyen H.S., Do D.T., Voznak M. (2016). Two-way relaying networks in green communications for 5G: Optimal throughput and trade-off between relay distance on power splitting-based and time switching-based relaying SWIPT. AEU-Int. J. Electron. Commun..

[3] Nguyen H.S., Bui A.H., Do D.T., Voznak M. (2016). Imperfect channel state information of AF and DF energy harvesting cooperative networks. China Commun..

[4] Chu Z., Zhou F., Zhu Z., Hu R.Q., Xiao P. (2018). Wireless Powered Sensor Networks for Internet of Things: Maximum Throughput and Optimal Power Allocation. IEEE Internet Things J..

[5] Mekikis P.V., Lalos A.S., Antonopoulos A., Alonso L., Verikoukis C. (2014). Wireless Energy Harvesting in Two-Way Network Coded Cooperative Communications: A Stochastic Approach for Large Scale Networks. IEEE Commun. Lett..

[6] Sudevalayam S., Kulkarni P. (2011). Energy Harvesting Sensor Nodes: Survey and Implications. IEEE Commun. Surv. Tutor..

[7] Guo S., Wang F., Yang Y., Xiao B. (2015). Energy-Efficient Cooperative Tfor Simultaneous Wireless Information and Power Transfer in Clustered Wireless Sensor Networks. IEEE Trans. Commun..

[8] Fotino M., Gozzi A., Cano J.C., Calafate C., Rango F., Manzoni P., Marano S. (2007). Evaluating energy consumption of proactive and reactive routing protocols in a MANET. IFIP Int. Fed. Inf. Process..

[9] Mekikis P.V., Antonopoulos A., Kartsakli E., Lalos A.S., Alonso L., Verikoukis C. (2016). Information Exchange in Randomly Deployed Dense WSNs with Wireless Energy Harvesting Capabilities. IEEE Trans. Wirel. Commun..

[10] Wang C., Li J., Yang Y., Ye F. (2018). Combining Solar Energy Harvesting with Wireless Charging for Hybrid Wireless Sensor Networks. IEEE Trans. Mob. Comput..

[11] Kosunalp S. (2017). An energy prediction algorithm for wind-powered wireless sensor networks with energy harvesting. Energy.

[12] Prijić A., Vračar L., Vučković D., Milić D., Prijić Z. (2015). Thermal Energy Harvesting Wireless Sensor Node in Aluminum Core PCB Technology. IEEE Sens. J..

[13] Guo S., Wang C., Yang Y. (2014). Joint Mobile Data Gathering and Energy Provisioning in Wireless Rechargeable Sensor Networks. IEEE Trans. Mob. Comput..

[14] Perera T.D.P., Jayakody D.N.K., Sharma S.K., Chatzinotas S., Li J. (2018). Simultaneous Wireless Information and Power Transfer (SWIPT): Recent Advances and Future Challenges. IEEE Commun. Surv. Tutor..

[15] Nguyen H.S., Nguyen T.S., Voznak M. (2018). Relay selection for SWIPT: Performance analysis of optimization problems and the trade-off between ergodic capacity and energy harvesting. AEU-Int. J. Electron. Commun..

[16] Varshney L.R. Transporting information and energy simultaneously. Proceedings of the 2008 IEEE International Symposium on Information Theory.

[17] Zhou X., Zhang R., Ho C.K. (2013). Wireless Information and Power Transfer: Architecture Design and Rate-Energy Tradeoff. IEEE Trans. Commun..

[18] Nasir A.A., Zhou X., Durrani S., Kennedy R.A. (2013). Relaying Protocols for Wireless Energy Harvesting and Information Processing. IEEE Trans. Wirel. Commun..

[19] Peng C., Li F., Liu H. (2017). Wireless Energy Harvesting Two-Way Relay Networks with Hardware Impairments. Sensors.

[20] Mouapi A., Hakem N. (2018). A New Approach to Design Autonomous Wireless Sensor Node Based on RF Energy Harvesting System. Sensors.

[21] Le Q.N., Bao V.N.Q., An B. (2018). Full-duplex distributed switch-and-stay energy harvesting selection relaying networks with imperfect CSI: Design and outage analysis. J. Commun. Netw..

[22] Nguyen D.K., Jayakody D.N.K., Chatzinotas S., Thompson J.S., Li J. (2017). Wireless Energy Harvesting Assisted Two-Way Cognitive Relay Networks: Protocol Design and Performance Analysis. IEEE Access.

[23] Olofsson T., Ahlén A., Gidlund M. (2016). Modeling of the Fading Statistics of Wireless Sensor Network Channels in Industrial Environments. IEEE Trans. Signal Process..

[24] Zhao F., Lin H., Zhong C., Hadzi-Velkov Z., Karagiannidis G.K., Zhang Z. (2018). On the Capacity of Wireless Powered Communication Systems over Rician Fading Channels. IEEE Trans. Commun..

[25] Hu Y., Cao N., Chen Y. (2016). Analysis of Wireless Energy Harvesting Relay Throughput in Rician Channel. Mob. Inf. Syst..

[26] Mishra D., De S., Chiasserini C.F. (2016). Joint Optimization Schemes for Cooperative Wireless Information and Power Transfer over Rician Channels. IEEE Trans. Commun..

[27] Schenk T. (2008). RF Imperfections in High-Rate Wireless Systems: Impact and Digital Compensation.

[28] Bjornson E., Matthaiou M., Debbah M. (2013). A New Look at Dual-Hop Relaying: Performance Limits with Hardware Impairments. IEEE Trans. Commun..

[29] Nguyen T.N., Duy T.T., Luu G.T., Tran P.T., Voznak M. (2017). Energy Harvesting-based Spectrum Access with Incremental Cooperation, Relay Selection and Hardware Noises. Radioengineering.

[30] Katti S., Gollakota S., Katabi D. (2007). Embracing Wireless Interference: Analog Network Coding. SIGCOMM Comput. Commun. Rev..

[31] Qin J., Zhu Y., Zhe P. (2017). Broadband Analog Network Coding with Robust Processing for Two-Way Relay Networks. IEEE Commun. Lett..

[32] Zwillinger D., Moll V., Gradshteyn I., Ryzhik I., Zwillinger D. (2015). Table of Integrals, Series, and Products.

[33] Bhatnagar M.R. (2013). On the Capacity of Decode-and-Forward Relaying over Rician Fading Channels. IEEE Commun. Lett..

[34] McKay M.R., Grant A.J., Collings I.B. (2007). Performance Analysis of MIMO-MRC in Double-Correlated Rayleigh Environments. IEEE Trans. Commun..

[35] Duong T.Q., Duy T.T., Matthaiou M., Tsiftsis T., Karagiannidis G.K. Cognitive cooperative networks in dual-hop asymmetric fading channels. Proceedings of the 2013 IEEE Global Communications Conference (GLOBECOM).

[36] Chong E.K.P., Zak S.H. (2013). An Introduction to Optimization.

[37] Morosi C., Pizzocchero L. (2004). On the expansion of the Kummer function in terms of incomplete Gamma functions. Arch. Inequal. Appl..

